# Who is left out? A systematic review on the barriers and facilitators for screening participation reported by people in vulnerable situations with strategies for the future

**DOI:** 10.1016/j.puhip.2026.100803

**Published:** 2026-05-08

**Authors:** J. van der Kamp, L. Krabbenborg, L.M. Kieneker, A.F. de Winter

**Affiliations:** a Institute for Science in Society, Radboud University, Heyendaalseweg 135, 6500 GL Nijmegen, the Netherlands; b Division of Nephrology, Department of Internal Medicine, University Medical Center Groningen, University of Groningen, Groningen, the Netherlands; c Department of Community & Occupational Medicine, University Medical Centre Groningen, University of Groningen, Antonius Deusinglaan 1, 9713 AV, Groningen, the Netherlands

**Keywords:** Screening participation, People in vulnerable situations, Barriers and facilitators

## Abstract

**Objectives:**

This review aims to give a comprehensive overview of barriers and facilitators reported by people in vulnerable situations (PiVS) for screening participation. Furthermore, it investigates proposed strategies to increase screening participation, as reported in quantitative and qualitative empirical studies.

**Study design:**

Systematic review.

**Methods:**

The databases Embase, CINHAL, PsycINFO, Web of Science and PubMed including Medline were systematically searched for papers (from January 2013 to June 2023) on barriers and facilitators experienced by PiVS on participating in screening programmes on chronic diseases of high-income countries. The included papers were analysed thematically.

**Results:**

96 papers were included. Results show barriers and facilitators mostly related to health literacy e.g. lack of resources, feelings of anxiety e.g. regarding test outcomes, and feelings of doubt e.g. regarding Western healthcare practices. Factors influencing screening participation can act both as barriers and facilitators. Studies suggested education, community-based interventions and support from healthcare providers as strategies to increase screening participation. The results show a focus on ethnic minorities as study populations. These populations reported mainly socio-cultural barriers and facilitators, such as religious beliefs. Consequently, there is limited representation of people in diverse vulnerable situations.

**Conclusions:**

This paper represents the voices of PiVS by showing barriers and facilitators reported by themselves. Currently, there is limited representation of diverse vulnerable groups and limited insight into how barriers and facilitators play out in people's daily lives. Therefore, we propose for future research to study the everyday-life to gain in-depth insight into the intersection of barriers and facilitators and how they influence decision-making. We propose for future screening developers to use participatory practices to build upon the experiences of diverse PiVS in order to develop and implement more inclusive screening programmes.

## 1 Plain language summary - research overview: Why vulnerable groups do (not) participate in disease screening

This paper investigates: 1) why people in vulnerable groups do (not) participate in disease screening programmes for chronic diseases, such as chronic kidney disease, and 2) strategies proposed to stimulate participation in screening programmes. The authors searched five major databases for scientific studies. They focused on research from high-income countries that studied people in vulnerable groups and their experiences with screening programmes. 96 studies were selected. The main reasons why people in vulnerable groups do participate in screening were: 1) increased knowledge on screening and disease, and 2) encouragement by doctors, family members or peers. The main reasons why people in vulnerable groups do not participate in screening were: 1) lack of means and capabilities to participate, such as a lack of money to pay for disease related costs, 2) feelings of anxiety, for example regarding test outcomes that would indicate disease, and 3) feelings of doubt, for example regarding Western healthcare practices. Some reasons, such as religious beliefs or relation with doctors, were reported as why people participate and not participate, depending on the situation or person. To improve participation in screening programmes, studies suggested to educate people, set-up programmes in different communities and increase encouragement by doctors. Most studies focused on people who are part of ethnic minority groups, who often reported cultural reasons to (not) participate in screening. While important, other vulnerable groups were underrepresented. Therefore, the authors suggest participatory research for the future to involve a wider range of people and better understand how people make decisions on screening participation in their everyday lives.

## 1 Introduction

Researchers, policymakers and health care professionals (HCPs) develop population-level screening programmes with the aim to identify diseases in early stages, e.g. colorectal cancer screening. Screening programmes aim to improve prognosis, prevent further disease progression in individuals and lower economic burden on society [1]. This is especially important for diseases that are considered chronic, such as chronic kidney disease and cancer, because of the high onset, progression and multimorbidity rates of chronic diseases [2]. In response on the high burden of chronic diseases on individuals and society, scholarly attention for different screening routes, such as screening at home, has increased [3]. This paper is part of the research project Check@Home, which aims to develop a comprehensive home-based screening programme for cardiovascular disease, chronic kidney disease and type II diabetes, in the Netherlands. Through different modes of testing and comprehensive screening, research on the development of screening programs aim to increase citizen participation and early detection of multiple diseases [4]. Yet, research shows that people in vulnerable situations (PiVS) are often underscreened for many different screening programmes and diseases, due to multiple barriers on individual and system levels [1,[5], [6], [7]]. Examples are limited health literacy, experiences of racism or financial worries [7]. This is problematic when compared to the majority of citizens because PiVS are less likely to receive a timely diagnosis and treatment, resulting in disproportionate morbidity and mortality rates in PiVS [7].

We focus on barriers and facilitators articulated by PiVS for screening participation. Participants are often grouped together under varying expressions of vulnerability as a notion, e.g. low socioeconomic status, migration backgrounds or poverty. Yet, how vulnerability is understood has implications for the scientific knowledge and assumptions that are (re)produced. Labelling people as vulnerable risks stigmatizing people with particular backgrounds as vulnerable, while they do not have to be or feel vulnerable [8]. Vulnerability is shaped by multiple circumstances and relations between people and society, including structural and political processes that sustain inequity. By acknowledging vulnerability as intersectional, situational and structural, we speak of vulnerable life situations in this paper [9]. To take this broad definition of vulnerability into account, the search strategy includes both personal characteristics, e.g. socioeconomic disadvantage, and circumstances, e.g. living in deprived areas.

Medical researchers expect health outcomes to improve when the unique barriers of PiVS towards screening are identified and addressed for the development and implementation of inclusive screening programmes [7]. Therefore, we aim to identify underlying considerations formulated by PiVS themselves for participation in various screening programmes. Current reviews addressed factors influencing screening participation for specific PiVS e.g. ethnic backgrounds [5,6] or specific diseases e.g. colorectal cancer [1]. As far as we know there is no current overview of the barriers and facilitators for screening participation for chronic diseases of PiVS in high-income countries.^1^ This review fills this gap by providing a comprehensive overview of barriers and facilitators, reported in quantitative and qualitative empirical studies, reported by PiVS for participating in screening for chronic diseases of high-income countries. Moreover, it investigates suggested ways towards increasing screening participation.

## 2 Methods

This systematic review selected data following the PRISMA guidelines [10], after protocol registration on PROSPERO (CRD42023387049) accessible via https://www.crd.york.ac.uk/PROSPERO/view/CRD42023387049.

### 2.1 Search strategy

We performed a pilot-review (n = 6) to refine the search strategy and inclusion criteria. The search strategy consists of both free text, e.g. “disadvantaged group∗”, and MeSH terms when applicable, e.g. “Low Socioeconomic Status” [MeSH] (Appendix A). The search strategy was finalized in consultation with the university librarian. Embase, CINHAL, PsycINFO, Web of Science and PubMed including Medline were searched from January 2013 to June 2023.

### 2.2 Eligibility criteria

We included papers with original peer-reviewed empirical evidence on barriers and facilitators reported by adults (18+) in vulnerable situations for screening programmes on chronic diseases in high-income countries. We included studies in high-income countries, to focus on the specific context of screening programmes in high-income countries. As most high-income countries have implemented and organised screening programs with more resources compared to low- and middle-income countries [11]. In light of our broad definition of vulnerability, papers with different definitions of vulnerable groups were included. Papers were excluded when populations were not considered vulnerable e.g. perspectives from general practitioners (GPs). Non-original research (e.g. opinion pieces), studies without empirical evidence in which PiVS reported barriers and facilitators themselves or papers not published in English were excluded.

### 2.3 Data extraction

The following data was extracted: 1) methodology, 2) study population, 3) age, 4) sample size, 5) country, 6) type of screening that was (not) participated in, 7) barriers and facilitators for screening participation, 8) strategies to increase screening participation and 9) main conclusions.

### 2.4 Data analysis

The papers varied in study populations, methods (quantitative, qualitative and mixed methods) and reported barriers and facilitators for screening participation. Therefore, we performed a narrative synthesis according to the ESRC guidance [12]. We identified facilitators and barriers through a thematic analysis by coding each factor reported in studies systematically using ATLAS.ti23 software. We explored the relationships between the papers in study characteristics, methods and reported barriers and facilitators on which we will elaborate in the results below. Quality assessment was completed using the Mixed Methods Appraisal Tool (Appendix B) [13]. Following the ESRC guidance, we moved iteratively through these steps.

## 3 Results

### 3.1 Study selection

**Fig. 1:**
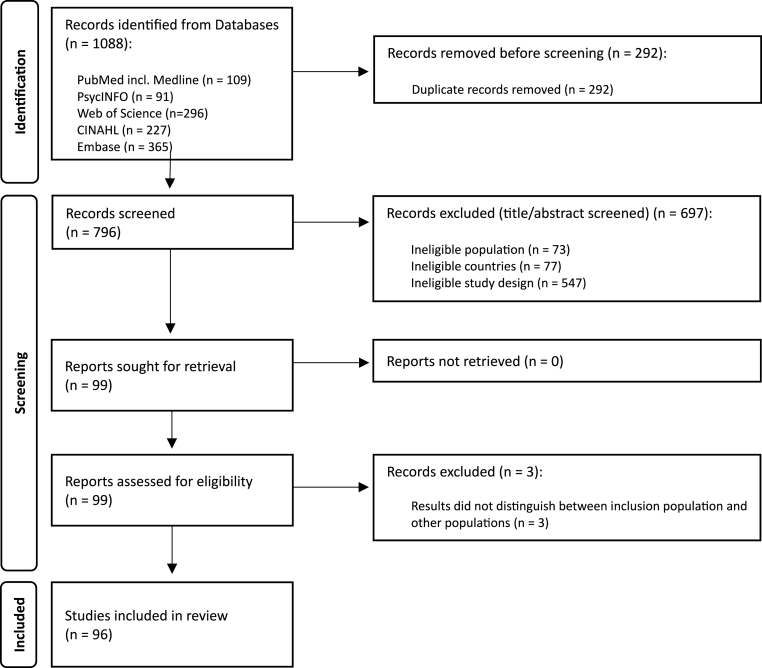
Preferred Reporting Items for Systematic Reviews and Meta-analyses (PRISMA) diagram.

The literature search selected 1088 papers and 796 remained after duplicates removal. We selected 99 papers based on title and abstract for full-text assessment. Full-text papers were assessed for eligibility based on the in- and exclusion criteria described above. Ultimately, 96 papers were included (Fig. 1).

### 3.2 Study characteristics

We included papers with a total of 44110 participants in varying study designs. We included 60 qualitative studies consisting of focus groups (29), interviews (17), both (14). 32 quantitative survey studies and 4 mixed methods studies combining qualitative and quantitative data. One study combined interviews, observations and document analysis. Geographically, studies were conducted in USA (60), Canada (9), UK (5), The Netherlands (4), Australia (3), Denmark, Norway (2), Belgium, Ireland, Portugal, Poland, Republic of Korea, Spain and Switzerland (1). The studies focussed on screening programmes for the following diseases; cancer (89), cardiovascular disease (1), diabetes (3) and hepatitis (3) (see Appendix B for details on the study characteristics).

We found that when it regards PiVS study populations were often linked to ethnic background since a majority (85%) of papers included people with migration backgrounds or from ethnic minorities (Appendix B). These groups reported mainly socio-cultural barriers. By defining vulnerability in terms of migration and ethnic minorities, there is a lack of insight into barriers and facilitators for other groups that are underscreened e.g. people living with low incomes (8) [[14], [15], [16], [17], [18], [19], [20], [21]], low-literacy (2) [15,22] or other illnesses (5) [17,[23], [24], [25], [26]].

### 3.3 Understanding barriers and facilitators for screening participation

The papers reported different barriers (Table 1) and facilitators (Table 2) for PiVS to participate in screening. We discuss barriers and facilitators in three themes: 1) health literacy, 2) feelings of anxiety, and 3) feelings of doubt.

**Table 1:** Overview of reported barriers for screening participation.

Barriers	# of papers				Papers
	Total	Qualitative method	Quantitative method	Mixed method	
**Health literacy**					

Lack of practical resources/access	61	39	19	3	[14,[16], [17], [18],^25^,[28], [84]]
Lack of knowledge/understanding	48	36	8	4	[15,16,[23], [24], [25], [26],^28^,^29^,[31], [32], [33], [34], [35], [36],^38^,^39^,^44^,^45^,^48^,^50^,[53], [54], [55],[59], [60], [61],[65], [66], [67], [68],[70], [71], [72], [73],^75^,^77^,[80], [92]]
Language barriers	34	28	4	2	[15,22,24,25,28,29,31,[33], [34], [35], [36],^38^,^39^,^45^,[50], [51], [52], [53], [54], [55],^58^,^59^,^61^,^62^,^67^,^71^,^73^,^74^,^79^,^80^,^83^,^90^,^91^,^93^]
Not wanting to know (until symptoms appear)	30	24	5	1	[18,[28], [29], [30], [31],^36^,^38^,^43^,^51^,[53], [54], [55],^59^,^62^,^64^,^65^,^68^,^69^,^71^,^74^,^75^,^77^,^79^,^88^,[90], [91], [92], [93], [94], [95]]
Belief that disease cannot be prevented	29	20	7	2	[17,29,30,32,35,41,43,49,54,55,57,62,64,65,[67], [68], [69],^71^,^77^,^82^,^86^,^88^,[91], [92], [93],[96], [97], [98]]
Low risk perception	25	18	6	1	[[28], [29], [30], [31],^35^,^36^,^39^,^43^,^46^,^49^,[53], [54], [55],^58^,^60^,^64^,^65^,^68^,^70^,^77^,^82^,^86^,^87^,^95^,^99^,^100^]
Difficulties with appointments	14	11	3	0	[18,24,25,31,33,36,66,69,71,77,78,82,83,96]
Lack of information that is considered necessary for participation	9	7	0	2	[26,31,32,62,71,78,84,91,100]
Lack of motivation/passiveness	7	5	2	0	[25,35,49,55,60,91,100]
Decisional balance	3	1	1	1	[25,26,81]

**Interactions with others and society**					

Socio-cultural customs and traditions	48	38	8	2	[15,16,25,[28], [29], [30], [31], [32], [33], [34],^36^,^37^,^39^,^41^,^43^,^45^,^50^,[52], [53], [54], [55],^57^,^58^,^62^,^64^,^65^,[68], [69], [70], [71],^75^,^77^,^78^,^80^,^83^,^84^,^86^,^88^,^90^,^91^,[93], [94], [95],[100], [103]]
Too occupied with daily challenges	38	29	7	2	[15,21,24,25,[28], [29], [30], [31], [32],^35^,^37^,^39^,^41^,^43^,[45], [46], [47],^50^,^51^,^54^,^55^,^59^,^62^,^63^,^66^,^69^,^70^,^74^,^75^,^77^,^78^,^82^,^83^,^85^,^86^,^95^,^96^,^99^]
Role of the GP/health provider	30	24	4	2	[24,25,28,29,[31], [32], [33],^38^,^51^,^54^,^58^,^60^,^61^,^68^,^70^,^72^,^74^,^75^,[77], [78], [79],^82^,^83^,^85^,^89^,^91^,^92^,[99], [100], [101]]
Shame/stigma	29	25	4	0	[15,25,28,31,[35], [36], [37],^39^,^41^,^44^,^45^,^48^,^50^,^53^,^55^,^58^,^72^,^77^,^83^,^85^,^86^,^89^,^90^,^93^,^95^,^96^,^99^,^100^]
Religious beliefs	25	20	3	2	[[28], [29], [30], [31], [32],^35^,[37], [38], [39], [40],^45^,[53], [54], [55],^62^,^64^,^68^,^77^,^83^,^85^,^86^,^88^,^92^,^93^,^99^,^104^]
Comparison to earlier experiences (of others)	19	16	2	1	[21,28,32,35,44,45,48,50,59,62,69,78,82,83,88,90,95,99,102]
Lack of support by family members	16	14	2	0	[25,31,33,39,41,43,45,55,58,62,65,70,83,86,90,92]
Not wanting to be a burden for others	2	2	0	0	[44,74]

**Feelings of anxiety**					

Anxious about possible outcomes	28	21	6	1	[15,18,21,25,[30], [31], [32],^39^,^41^,^43^,^44^,^46^,^50^,^53^,^55^,^59^,^66^,^67^,^69^,^82^,^83^,^85^,^90^,^91^,^93^,^95^,^99^,^105^]
Anxious about testing procedure	27	18	8	1	[16,17,25,29,39,41,43,46,48,49,55,62,[64], [65], [66], [67],^69^,[71], [72], [73],^84^,^88^,^91^,^92^,^95^,^102^,^106^]
Broad feelings of anxiety e.g. fear of disease, being pitied or being deported when having an illegal status	18	13	3	2	[15,21,26,28,30,32,35,44,45,55,62,68,72,78,91,92,102,107]
Anxious about possible treatment	3	3	0	0	[48,55,99]

**Feelings of doubt**					

Doubting the (Western) health system	32	27	2	3	[23,25,28,29,[31], [32], [33], [34], [35],^37^,^38^,^41^,^43^,^45^,^46^,^48^,^50^,^51^,^54^,^55^,^65^,[67], [68], [69], [70], [71],^73^,^83^,^89^,^90^,^92^,^93^]
Doubting effectiveness of the test	18	15	3	0	[34,38,39,43,51,59,63,67,74,77,82,83,85,88,90,93,103,108]
Doubting usefulness of preventative screening/care	11	10	0	1	[31,32,59,67,71,74,83,88,90,93,99]
Doubting own ability to perform self-tests	6	5	1	0	[38,44,63,77,92,108]
Doubting effectiveness of treatment	6	5	0	1	[32,45,68,71,82,90]

Only barriers that were reported as findings of the papers are included in this table.

**Table 2:** Overview of reported facilitators for screening participation.

Facilitators	# of papers				Papers
	Total	Qualitative method	Quantitative method	Mixed method	
**Health literacy**					

Education/knowledge	28	13	13	2	[[15], [16], [17],^26^,^33^,^34^,^39^,^40^,^43^,^49^,^54^,^60^,^66^,[68], [69], [70],^74^,^79^,^84^,[96], [97], [98],^101^,^103^,^104^,[107], [108], [109]]
Perceived benefits of screening	18	12	6	0	[12,22,29,34,39,40,46,48,49,53,77,[85], [86], [87],[97], [98], [99],^101^]
Access to health care	18	7	11	0	[17,19,23,34,39,40,51,57,61,64,66,69,71,79,81,94,97,104]
Low effort/accessible screening modalities	14	10	4	0	[19,20,34,38,44,48,56,63,71,78,91,101,104,108]
Feeling responsible for the protection of good health	13	10	3	0	[15,23,30,33,41,62,69,85,87,93,94,104,110]
Reassurance about one's health status	9	7	1	1	[25,32,49,51,53,74,75,85,91]
Avoid disease progression	9	7	0	2	[15,30,32,44,53,54,84,91,101]
Avoid intense treatment	3	3	0	0	[30,53,74]
Perceived severity of disease	7	5	2	0	[15,34,52,53,57,68,104]
Perceived susceptibility of disease	7	3	3	1	[26,66,75,91,92,98,104]
Reduced health care costs	2	2	0	0	[38,85]
Decisional balance	2	0	1	1	[26,109]

**Interactions with others and society**					

Encouragement by GP/health provider	32	22	8	2	[17,18,25,26,30,[34], [35], [36],^40^,^44^,^54^,^59^,^64^,^66^,[68], [69], [70], [71],^74^,^75^,^77^,^79^,^81^,^87^,^91^,^92^,[98], [99], [100],^102^,^109^,^110^]
Encouragement by family/friends/peers	27	19	5	3	[15,25,26,30,32,35,48,54,56,67,69,70,74,79,[83], [84], [85], [86],^90^,^91^,^93^,^96^,^99^,^102^,^104^,^108^,^110^]
Trust in health care (provider)	16	15	0	1	[15,23,25,36,44,54,62,[67], [68], [69],^74^,^75^,^77^,^83^,^90^,^100^]
Comparison to earlier experiences (of others)	15	12	3	0	[34,35,43,47,50,57,70,74,75,77,78,85,91,99,106]
Religion as support	12	11	0	1	[30,32,33,43,53,69,85,86,88,89,92,93]
Privacy when self-sampling	4	3	1	0	[19,38,77,78]
Civic duty	2	2	0	0	[51,85]

Only facilitators that were reported as findings of the papers are included in this table.					

Related to health literacy we found individual, social and systematic determinants that affect how ‘health-related information is accessed, obtained, processed, understood and utilized’ (p.110) [27]. More than half (64%) of the papers reported lack of practical resources/access, e.g. means to cover healthcare costs, a barrier for screening participation. 11 out of 22 quantitative studies reported lack of practical resources/access as statistically significant. This was linked to practical barriers e.g. language barriers (34) or difficulties in appointments (14). It was also linked to the Western degree of preventative care not always aligning with socio-cultural customs and traditions e.g. preferring alternative medicine when symptomatic or women not exposing their bodies to male HCPs. As a facilitator, 18 papers reported access to health care as a facilitator (7 out 11 statistically significant). 14 papers (1 out of 4 statistically significant) reported low effort/accessible screening modalities, e.g. free, home-based or multi-disease testing, as facilitators.

Another barrier in the health literacy theme that half (50%) of all papers reported was lack of knowledge/understanding (4 out of 12 statistically significant). A paper on cancer screening uptake of low-income and illiterate immigrant women in France showed for example that people had difficulty understanding screening invitation letters which resulted in them discarding the letter without participating [15]. 30 papers (4 out of 6 statistically significant) showed not wanting to know (until symptoms appear) as a barrier. We found that how people value health(care) and their body has influence on screening participation. Papers that studied people with migration backgrounds or from ethnic minorities reported that in some cultures people only engage with healthcare once bodily symptoms are experienced. This is opposed to the concept of screening which is based on preventative care when asymptomatic. Different values related to screening emerged which are influenced by the belief that a disease cannot be prevented (29) and low risk perceptions (25).

Half (50%) of all papers reported socio-cultural customs and traditions, e.g. talking about disease is not commonplace in some communities, as barriers for screening participation (2 out of 10 statistically significant). Another barrier was being too occupied with daily challenges, e.g. home or work duties, as reported by 38 papers (3 out of 9 statistically significant). For example, a study on immigrant women's barriers and facilitators to cervical cancer screening reported that women prioritized taking time for financial problems in their families over their personal health, including preventative behaviour such as screening participation [28].

It is notable that some factors, e.g. religious beliefs or the role of HCPs, can act as both barriers and facilitators. For example, a study on immigrant women's barriers to cervical cancer screening reports that women avoid screening because of the importance of God's will on one's life [29]. While another paper on colorectal cancer screening perceptions of Korean Americans reported religion as a support for screening because the body is a gift from God and therefore one's responsibility to care for it [30]. Another example of duality is the role of the GP. The third most (31%) reported barrier (2 out of 6 statistically significant) was the role of the GP/health provider. For instance because people did not feel taken serious by their HCPs e.g. because of (unconscious) biases of GPs regarding skin colour or female genital mutilation [[31], [32], [33]]. While as a facilitator, encouragement by GP/health provider was reported the most (32 papers, 7 out of 10 statistically significant), e.g. because GPs were considered reliable [[34], [35], [36]].

In the second theme, we found that anxiety was often reported: 28 papers reported people feeling anxious because of possible outcomes from the screening, e.g. being diagnosed, as a barrier for participation (1 out of 7 statistically significant). Another reported cause of anxiety was the testing procedure itself (27 papers, 3 out of 9 statistically significant), e.g. expected pain during mammography.

In the third theme, regarding feelings of doubt, most papers (32) reported people doubting the (Western) health system, e.g. distrusting abilities of HCPs (1 out of 5 statistically significant). Doubting the effectiveness of the tests, e.g. regarding false-positive or false-negative results, was also reported as a barrier for screening participation in 18 papers (1 out of 3 statistically significant).

While the second and third themes did not include facilitators, encouragement by health providers or family/friends/peers, trust in health care (providers) and support from religion (reported by 32, 27, 16 and 12 papers, respectively), could reduce feelings of anxiety or doubt. Yet, we found a lack of inquiry into how barriers and facilitators influence each other and how social contexts, e.g. daily life with family or work, influence screening participation. Barriers and facilitators can be influenced by values that are prominent in different communities at different times. For example, for the barrier ‘a lack of practical resources/access’, one study on barriers for low-income women showed that financial barriers include healthcare and related costs e.g. lost pay when taking time off or paying for childcare in order to get screened [14]. Another study on immigrant people's perceptions of prostate cancer screening showed that many are working for better futures for their families and want to avoid spending money on health insurance. This resulted in them not participating in screening to avoid possible healthcare costs [37].

### 3.4 Suggested ways towards increasing screening participation

The authors of included papers suggested strategies to increase participation in future screening programmes in the results, discussion or conclusion sections (Table 3). More than half (61%) of the papers suggested education to overcome barriers. While some papers performed intervention studies to test education strategies, others did not show empirical results on the effectiveness of strategies to increase screening participation. For example, one paper studied education as an intervention to resolve misconceptions of breast cancer screening among immigrant women. The intervention consisted of culturally sensitive face-to-face education. As a result, cancer knowledge and perceived screening benefits improved with statistical significance [65]. Yet, another study argues that people do not engage with health education because of disease-related stigma present in some communities. Therefore, the authors discuss the need for community-based interventions instead of educational campaigns [86]. This can address a diverse intersection of barriers e.g. lack of support from the community and religious beliefs. Many studies highlighted community-based interventions (39), increase access/low effort screening (37), support from HCPs (43) or a combination of these strategies as promising to increase screening participation. Examples are cooperations between researchers and community health workers [65], home-based testing or mobile services [20,87,96] and encouragement to participate in screening from HCPs [24].

**Table 3:** Suggested strategies to increase screening participation.

Suggested strategies	Example	# of papers	Papers
**Individual**			

Educate citizens	Education programmes	59	[14,17,20,21,23,24,26,28,[31], [32], [33], [34],^36^,^38^,^39^,[43], [44], [45],^49^,^50^,^54^,^55^,[57], [58], [59], [60],[64], [65], [66],[68], [69], [70], [71], [72], [73], [74], [75], [76], [77], [78],^81^,^84^,^90^,^91^,[93], [97],[100], [102],[104], [107],^109^,^110^]
Decrease stigma	Health provider should listen and ask questions	13	[29,35,53,55,59,71,78,81,86,89,92,99,104]
Decrease mistrust	Health provider should convey care and respect to foster mutual trust	11	[15,28,32,35,38,45,54,55,64,65,71]
Empower people to make health decisions	Empower through education by peers	11	[17,21,41,43,57,71,75,77,95,98,109]
Decrease anxiety	Include stories about survival instead of focussing on disease	6	[21,26,36,64,70,77]

**Community**			

Community-based interventions	Involve trusted community-based organisations to promote screening	39	[20,23,24,28,31,33,[35], [36], [37], [38], [39],[42], [43], [44], [45],^47^,^49^,^50^,^54^,^58^,^60^,^63^,^65^,^68^,^69^,^71^,[73], [74], [75],^81^,^83^,^86^,^87^,^92^,^93^,^95^,^101^,^104^,^108^,^110^]
(Religious) role models	Involve key figures in the community to promote screening	29	[24,28,32,33,35,[39], [40], [41],^43^,^45^,^48^,^49^,^53^,^55^,^67^,^68^,^70^,^71^,^74^,^77^,^83^,^88^,[91], [92], [93],^95^,^103^,^106^,^108^]
Targeted strategies per group	Tailored outreach towards particular ethnic and cultural groups	16	[22,24,26,36,65,68,72,82,[90], [91], [92],^95^,^97^,^98^,^107^,^108^]
(Stimulate) support from peers/family/friends	Give attention to people's social network	14	[25,30,32,50,58,59,69,77,86,88,89,92,96,108]

**System**			

Support from/access to health provider	Involve general practitioners to promote screening	43	[17,18,21,[23], [24], [25], [26],^28^,^30^,^31^,[33], [34], [35], [36],^39^,^54^,[59], [60], [61],[63], [64], [65],[67], [68], [69], [70], [71],[73], [74], [75],^77^,^81^,^84^,^85^,^87^,^89^,^92^,^95^,^97^,[99], [100], [101],^108^,^109^]
Increase access/low-effort screening	Home test kits or mobile units	37	[17,[19], [20], [21],^24^,^28^,^29^,^35^,^39^,^41^,^42^,[44], [45], [46],[50], [51], [52],^54^,^56^,^59^,[62], [63], [64], [65], [66],^70^,^72^,^73^,^77^,^78^,^85^,^86^,[95], [96], [97],^99^,^107^]
Educate health providers	Educate health providers on potential attitudes, beliefs and worries	27	[15,20,26,28,29,31,33,35,36,40,41,43,[50], [51], [52],^64^,^68^,^69^,^74^,^75^,^77^,^84^,^87^,^89^,^93^,^94^,^106^]
Patient-provider gender concordance	During gender sensitive screening tests e.g. when it is necessary to be undressed	13	[28,29,32,33,36,38,71,84,91,93,96,101,102]
Multi-level strategies	Combine education, improved access and screening promotion	9	[17,21,29,35,64,65,74,102,108]
Free screening/financial assistance	Reduce burden of nonmedical costs related to screening	9	[14,35,36,38,54,69,70,79,97]
Facilitate follow-up treatment/care	Give advise on navigating the complex health care system	5	[31,48,59,63,79]

**Communication**			

Clear information materials/communication	Avoid jargon	31	[17,19,[21], [22], [23],^28^,^30^,[34], [35], [36],^38^,^41^,^44^,^53^,^58^,^59^,^67^,^70^,^71^,^76^,^77^,^81^,^84^,^85^,^92^,^93^,^99^,^102^,[106], [107], [108],^110^]
Multilingual information (materials)	Translate information documents into multiple languages	26	[22,24,28,29,33,34,51,52,55,58,59,65,67,69,73,75,77,79,87,91,92,96,100,102,108,110]
Advertisement/awareness campaign	Posters and TV advertisements	26	[23,24,31,[33], [34], [35],^44^,^50^,^54^,^59^,^64^,^67^,^69^,^70^,^74^,^75^,^77^,^79^,^85^,^89^,^91^,^92^,^95^,^97^,^99^,^100^]
Reminder after invitation letter	Through mobile text message	17	[15,18,21,24,25,28,34,39,44,62,69,70,76,82,91,100,108]
(Culture) sensitive writing	Avoid judgement and show respect for cultural differences	14	[21,31,32,37,44,55,68,69,77,84,89,91,97,108]
Promotion via online platforms	Messages via social media	10	[24,28,34,38,39,76,79,102,108,110]
Develop screening (information) with end-users	Include communities to develop culturally sensitive interventions	9	[15,21,24,44,45,64,89,97,110]
Visual information materials	Support text with visuals such as pictures	8	[22,26,33,67,71,91,92,108]
Facilitate a space for questions	Phone number to call	4	[44,70,76,108]

The strategies included in this table were suggested by the authors in the results, discussion or conclusion sections of the papers.			

## 4 Discussion

This review presented barriers and facilitators to participate in screening for PiVS and strategies to increase screening participation. We found five main results: 1) barriers were related to health literacy, feelings of anxiety and doubt, 2) facilitators were related to health literacy, interactions with others (e.g. family and HCPs), 3) factors influencing screening participation can act both as barriers and facilitators, 4) mostly ethnic minorities were included as study groups, and 5) possible strategies to increase screening participation for PiVS.

First, barriers were mostly related to 1) health literacy including (lack of) knowledge, resources or social support and religious beliefs; 2) feelings of anxiety for instance regarding test outcomes, and 3) feelings of doubt e.g. regarding Western healthcare practices. Second, facilitators were primarily related to 1) health literacy such as knowledge, and 2) interactions with others and society, e.g. encouragement by HCPs. These findings reflect those of earlier research, which showed that knowledge on the disease, healthcare access such as costs, culture e.g. religious beliefs and social interactions influence screening participation [5,111].

Third, factors influencing screening participation can act as both barriers and facilitators, which was also shown by a systematic review which did not focus on vulnerable groups [112]. This duality is important to take into account when designing interventions that effectively promote screening participation and address contextual differences.

Fourth, the literature regarding PiVS focusses on ethnic minorities as study populations who reported mainly socio-cultural barriers and facilitators. While important, this also shows that other PiVS are less studied which means that other barriers and facilitators for screening participation remain less visible. The classification of study populations is needed to gain insight into specific needs of different groups. Yet, selective study populations have consequences for whose needs become visible or remain invisible and which strategies to increase screening participation are effective [113].

Fifth, many studies suggested education, community-based interventions, increasing access to screening, support from HCPs or a combination of these strategies to increase screening participation. These strategies were considered promising in previous research [5,7,114]. Yet, increasing screening participation is not clear-cut because of the duality of factors that influence participation and the lack of representation of diverse vulnerable groups. To overcome these gaps, implications for future research and practice are given below to support researchers, policy makers and HCPs to better understand, develop and implement inclusive screening.

### 4.1 Strengths and limitations

This systematic review provides a comprehensive overview of barriers and facilitators that influence screening participation of PiVS. By including papers with empirical evidence on barriers and facilitators that PiVS reported themselves, this review represents the voices of PiVS which are often overlooked in research. Yet, this methodological approach is limited in three ways. First, through our inclusion criterium of barriers and facilitators reported by PiVS themselves, we did not include randomized control trials (RCTs) which could have given insight into the effectiveness of strategies to increase screening participation. For example, another review on intervention studies, e.g. RCTs, for screening participation of women with migration backgrounds, showed interventions that address logistical barriers and communicate culturally sensitive information as effective [114]. Second, papers written in languages other than English or studies performed in non high-income countries were not included. Therefore, there might be other barriers, facilitators and experiences regarding screening participation that we did not cover. Third, while we investigated barriers and facilitates reported by PiVS themselves, we did not cover barriers and facilitators of people who are not considered PiVS. For example, those who intentionally not participate because of moral beliefs [115].

### 4.2 Implications for future research and practice

Through our results, we identified two main gaps. First, several PiVS are currently underrepresented in research, e.g. people living with low incomes or low-literacy, which may mean that their needs are overlooked in policy and healthcare decision-making. The studies in this review, used methods that required cognitive, linguistic and deliberative abilities from participants, e.g. interviews, which do not always match what PiVS are comfortable with [116,117]. As argued by Raap et al. (2024) research methods influences who can(not) participate, shaping how public health issues are defined in research and practice [116]. We therefore argue that future research should investigate everyday lives of PiVS to uncover the social context in which 1) intersections between barriers and facilitators influence people's lives, which remain understudied when barriers and facilitators are considered as stand-alone; and 2) people make decisions regarding their health and participation in screening. To investigate the everyday-life, future research should employ different methodologies beyond the often utilized focus groups, interviews or surveys. Pols (2023) argues for researchers engaging in activities with people less able to voice their needs, as a promising method for understanding the everyday-life [117]. Second, participatory practices are promising, as it provides opportunities for developers to include the needs and preferences of diverse PiVS in every stage of development and implementation of screening to contribute to more inclusive screening programmes [118].

## Ethical statement

No ethical approval was required for this review article since the research uses existing data.

## Funding

This publication is part of the Check@Home project with project number KICH2.V4C.20.005 of the research program Knowledge and Innovation Covenant - DEMAND 2020 which is partly financed by the Dutch Research Council (NWO), and partly by the Dutch Heart Foundation, the Dutch Kidney Foundation, the Dutch Diabetes Research Foundation, 10.13039/100004325AstraZeneca BV, 10.13039/100016545Roche Diagnostics, 10.13039/501100011699Siemens Healthineers Netherlands BV, Happitech BV, and Topicus Healthcare BV.

## Declaration of competing interest

The authors declare the following financial interests/personal relationships which may be considered as potential competing interests:Our research was conducted independently; the private partners disclosed in the funding statement had no influence on the design, analysis or outcomes of our research as reflected in the consortium agreement. The authors have no other conflicts of interest to disclose.

## Footnotes

1 High-income countries based on the New World Bank country classification (2022).
