# Supplementary material for: Who is left out? A systematic review on the barriers and facilitators for screening participation reported by people in vulnerable situations with strategies for the future
Source: Public Health Pract (Oxf). 2026 May 8;11:100803. doi: 10.1016/j.puhip.2026.100803 (PMC13194543; doi:10.1016/j.puhip.2026.100803)
Supplement: Multimedia component 1 [file mmc1.docx]

# Appendix B: Characteristics of included studies (96)

| **Author (Year)** | **Title** | **Method** | **Study population** | **Age** | **Sample size** | **Country** | **Type of screening that was (not) participated in** | **Main conclusion (cited)** | **MMAT** ^^[[1]](#footnote-1)^^ |
| --- | --- | --- | --- | --- | --- | --- | --- | --- | --- |
| Adegboyega et al. (2022) | Qualitative assessment of attitudes toward cervical cancer (CC) screening and HPV self-sampling among African American (AA) and Sub Saharan African Immigrant (SAI) women | Focus groups | Migration background/ethnic minority | 33.67± 9.03 | 30 | USA | Cervical cancer screening | 'This study provided evidence that AA/SAI women continue to experience barriers to CC screening which may deter routine and timely screening.' (p. 1781). | 4 |
| Akhagba (2017) | Migrant women's knowledge and perceived sociocultural barriers to cervical cancer screening programme: A qualitative study of African women in Poland | Focus groups | Migration background/ethnic minority | 25-54 | 12 | PL | Cervical cancer screening | 'This study explored the prevailing sociocultural issues affecting migrant women’s health in migration, which seem to have a great influence on acceptance and adoption of cervical cancer screening because they face everyday life difficulties such as linguistic, sociocultural and systematic difficulties preventing them from undertaking cervical cancer screening.' (p. 269) | 3 |
| Alam et al. (2022) | Cervical screening uptake: A cross-sectional study of self-reported screening attitudes, behaviours and barriers to participation among South Asian immigrant women living in Australia | Survey | Migration background/ethnic minority | 20-75 | 148 | AU | Cervical cancer screening | 'Factors related to low uptake such as recent arrival to Australia, lack of free and regular healthcare access and lack of language friendly resources need to be taken into account for this respective group, along with the perspectives of community collaborators and healthcare providers for successful intervention implementation.' (p. 10) | 3 |
| Allen et al. (2019) | Facilitators and Barriers of Cervical Cancer Screening and Human Papilloma Virus Vaccination Among Somali Refugee Women in the United States: A Qualitative Analysis | Focus groups | Migration background/ethnic minority | 23-64 | 31 | USA | Cervical cancer screening | 'Given the limited knowledge of HPV in this population, targeted intervention strategies that focus on increasing HPV vaccination among Somali men and women may have an impact on reducing cervical cancer disparities in this population.'(p. 61) | 4 |
| Ayash et al. (2020) | Associations with the Receipt of Colon Cancer Screening Among a Diverse Sample of Arab Americans in NYC | Survey | Migration background/ethnic minority | 59.96±7,26 | 100 | USA | Colorectal cancer screening | 'In this sample of 100 Arab Americans living in New York City, associations with the receipt of CRC screening uptake included socioeconomic factors (i.e. education level, health insurance coverage), doctor recommendations, period of residence in the United States, and spiritual and religious beliefs.' (pp. 508-509) | 3 |
| Baezconde-Garbanati et al. (2013) | Reducing the Excess Burden of Cervical Cancer Among Latinas: Translating Science into Health Promotion Initiatives | Focus groups | Migration background/ethnic minority | 21-45 | 97 | USA | Cervical cancer screening | 'Doctors and health educators may benefit from this research by having a better understanding the issues Latinas face. Persistent barriers identified in this study included: cost, lack of knowledge, lack of English proficiency, worries about cost of coming in for screening, and time constraints.' (p. 10) | 3 |
| Balata et al. (2019) | Attendees of Manchester's Lung Health Check pilot express a preference for community-based lung cancer screening | Survey | Deprived areas | 65.6±5.4 | 938 | UK | Lung cancer screening | 'In conclusion our results suggest that convenient community-based screening programmes, using mobile CT scanners, may be one approach to reduce inequalities in screening uptake and adherence especially in those at high risk of lung cancer in deprived areas.' (p. 1178) | 3 |
| Bea et al. (2023) | Sister, Give Me Your Hand: a Qualitative Focus Group Study on Beliefs and Barriers to Mammography Screening in Black Women During the COVID-19 Era | Focus groups | Migration background/ethnic minority | 46-73 | 33 | USA | Breast cancer screening | ' Our fndings on factors that afect breast cancer screening decisions point to both barriers and facilitators that are not solely related to poverty and insurance. Community outreach eforts should concentrate on building trust, providing equitable digital access, and skillfully addressing breast health perceptions while incorporating strategies to address fear and fatalism.' (p. 1476) | 5 |
| Bhargava et al. (2022) | Polish immigrants' access to colorectal cancer screening in Norway - a qualitative study | Interviews | Migration background/ethnic minority | 52-59 | 10 | NO | Colorectal cancer screening | 'In a transnational setting with substantial migration between Poland and Norway, we identifed several factors that could infuence Polish immigrants’ accessibility to the Norwegian CRC screening programme. In order to reduce morbidity and mortality from CRC, measures to improve accessibility for Polish immigrants should target these factors. Such measures include increasing cultural competence among HCPs and providing information in Polish through Polish-speaking health care professionals, GPs and internet portals used by the Polishspeaking community in Norway.' (p. 12) | 5 |
| Biddell et al. (2021) | Perceived financial barriers to cervical cancer screening and associated cost burden among low-income, underscreened women | Survey | Low-income | 25-64 | 702 | USA | Cervical cancer screening | 'Our findings highlight the nature of perceived financial barriers to, and cost burden of, cervical cancer screening in a particularly at-risk population. The majority of low-income women overdue for cervical cancer screening in our sample perceived financial barriers associated with the cost of the clinic appointment and potential future treatment costs.' (p. 1249) | 4 |
| Brown et al. (2022) | Increasing uptake to a lung cancer screening programme: building with communities through co-design | Focus groups and interviews | Deprived areas | 51-86 | 39 | UK | Lung cancer screening | 'the insights of this study led to three key recommendations to improve uptake to a future lung cancer screening programme amongst those who could beneft most: 1. Embedding the service in communities, 2. Efective communication and 3. Overcoming barriers with options. Tis along with the fndings regarding the acceptability of an associated biorepository should aid the development of an acceptable and efective future lung cancer screening programme, meeting the needs of those who could beneft most. Using co-design to further develop this programme will help to ensure that lung cancer screening will reach those who could beneft most.' (p. 15) | 4 |
| Casanova et al. (2021) | Harvesting Health Knowledge: Breast Cancer Perceptions in the South Florida Latinx Farmworker Community | Focus groups and interviews | Migration background/ethnic minority | 20-74 | 21 | USA | Breast cancer screening | 'This study elucidates the understandings and access barriers around breast cancer in the South Florida Latinx farmworker community across the following themes: lack of information, social and economic barriers, cultural factors, fear and mistrust, and psychosocial concerns.' (p. 1432) | 4 |
| Catarino et al. (2016) | Barriers to Cervical Cancer Screening in Geneva (DEPIST Study) | Survey | Migration background/ethnic minority | 25-69 | 556 | CH | Cervical cancer screening | 'Our findings support the hypothesis that the main reasons for nonparticipation in CC screening are practical barriers, such as lack of time and the cost of screening, and suggest that by overcoming these practical barriers, we can maximize participation in cervical screening.' (p. 138) | 3 |
| Costas-Muniz et al. (2020) | Colorectal Cancer Screening and Access to Healthcare in New York City Taxi Drivers | Survey | Nonstandard work arrangements | 50+ | 137 | USA | Colorectal cancer screening | 'The results suggest that, in a sample of male taxi drivers eligible for CRCS, poor access to primary care services (lack of insurance, not having a primary care provider and not visiting the provider within the past year) and occupation-related factors, such as working long hours (61 or more), and working a night shift, are key factors associated with reduced access to CRCS.' (p. 532) | 3 |
| Crawford et al. (2015) | A Peer Health Educator Program for Breast Cancer Screening Promotion: Arabic, Chinese, South Asian, and Vietnamese Immigrant Women's Perspectives | Focus groups and interviews | Migration background/ethnic minority | 40+ | 82 | CA | Breast cancer screening | 'The four main themes provided insights into immigrant women’s perceptions of the program including learning about breast health and prevention, access to social support, perceptions of screening and health services, and ways to improve programming.' (p. 10) | 4 |
| Crowley et al. (2018) | Barriers and facilitators to hepatitis C (HCV) screening and treatment-a description of prisoners' perspective | Focus groups | Prisoners | N/A | 46 | IE | Hepatitis screening | 'Irish prisons are a key setting to identify and treat HCV infected PWID. This important public health strategy can only be achieved by the elimination of identified barriers to HCV screening and treatment in Irish Prisons. The availability of short-acting, tolerable and highly effective DAA can eliminate many of these barriers but effective education programs highlighting the benefits of these treatments are required.' (p. 8) | 5 |
| Cullerton et al. (2016) | Cancer screening education: can it change knowledge and attitudes among culturally and linguistically diverse communities in Queensland, Australia? | Survey | Migration background/ethnic minority | 18+ | 146 | AU | Colorectal, breast and cervical cancer screening | 'The pilot cancer screening sessions appear to have enhanced positive attitudes toward cancer screening and increased intentions to participate in cancer screening among selected CALD communities. The culturally-tailored sessions delivered by MHWs in the appropriate languages, provided opportunities for community members to ask questions, and clarify information in a culturally safe environment. This is particularly important for community members with low levels of literacy. Tailoring of health information provided by health professionals is crucial for the appropriate delivery of health information to CALD communities.' (p. 146) | 3 |
| De Jesus et al. (2021) | Addressing Cancer Screening Inequities by Promoting Cancer Prevention Knowledge, Awareness, Self-Efficacy, and Screening Uptake Among Low-Income and Illiterate Immigrant Women in France | Interviews | Low-income and low-literate immigrant women | 25 - 75 | 164 | FR | Colorectal, breast and cervical cancer screening | 'The study findings suggest that addressing cancer prevention requires distinct approaches rather than a one-size-fits-all approach, taking into account the differences in levels of literacy, income, health knowledge, awareness, self-efficacy, and perceived barriers.' (p. 10) | 3 |
| Dharni et al. (2017) | Factors influencing participation in colorectal cancer screening-a qualitative study in an ethnic and socio-economically diverse inner city population | Interviews | Migration background/ethnic minority | 55-74 | 50 | UK | Colorectal cancer screening | 'Our findings suggest that there were considerable commonalities in the views of a diverse range of south London residents about some aspects of CRC screening, particularly awareness and the perceived benefits of screening. However, lower SES individuals wanted greater explanation of how to perform the test. Furthermore, our findings also demonstrate that perceptions of CRC screening are shaped by a multitude of psychological factors and lived experiences, which vary greatly between individuals.' (p. 616) | 4 |
| Ekeh (2022) | Health beliefs as predictors of intentions toward prostate cancer screening among Nigerian immigrant men | Survey | Migration background/ethnic minority | 40+ | 130 | USA | Prostate cancer screening | 'The findings of this study indicate that perceived susceptibility to prostate cancer and perceived benefits of prostate cancer screening significantly predicted NIM’s intentions to undergo prostate cancer screening.' (p. 769) | 3 |
| Fang & Baker (2013) | Barriers and Facilitators of Cervical Cancer Screening among Women of Hmong Origin | Focus groups | Migration background/ethnic minority | 18-61 | 44 | USA | Cervical cancer screening | 'Development of trust and a caring attitude on the part of all personnel involved in the health care delivery system can improve the willingness of Hmong women to be screened. This will require inclusion of Hmong cultural brokers as consultants for delivery systems and changes in the amount of reimbursable time for offi ce visits requiring interpreters' (p. 551) | 4 |
| Farr et al. (2021) | Stakeholder perspectives on colonoscopy navigation and colorectal cancer screening inequities | Observations, document analysis and interviews | Low-income and Migration background/ethnic minority | mean: 55 | 10 | USA | Colorectal cancer screening | 'Main themes emerging from the data displayed specific challenges faced by low-income Black populations and the value of the patient navigation from the perspectives of patients, providers and staff at the clinical site, and patient navigators. Patient participants demonstrated multiple categories of barriers to screening completion.' (p. 674) | 2 |
| Fletcher et al. (2014) | Perceptions of barriers and facilitators to cervical cancer screening among low-income, HIV-infected women from an integrated HIV clinic | Focus groups | Low-income and living with (other) illness | 26-61 | 33 | USA | Cervical cancer screening | 'Study findings support the need to employ a holistic approach to HIV/AIDS care which includes cancer preventive services. Existing HIV clinics which specialize in delivering comprehensive services can function as models for other HIV care centers by working to strategically integrate cancer preventive services into their current practices.' (p. 8) | 4 |
| Gathirua-Mwangi et al. (2018) | Factors associated with adherence to mammography screening among insured women differ by income levels | Survey | Low-income (compared to middle and high income) | 41-75 | 1642 | USA | Breast cancer screening | 'Our study shows differences in predictors of mammography adherence for different income groups. In multiple regression models, although age and a doctor’s recommendation were the strongest predictors of mammography adherence in the lowincome group, age and barriers were significant predictors in the high-income group.' (p. 467) | 4 |
| Gauss et al. (2013) | Pap screening goals and perceptions of pain among black, Latina, and Arab women: steps toward breaking down psychological barriers | Survey | Migration background/ethnic minority | 21-70 | 420 | USA | Cervical cancer screening | 'Perceived pain from the Pap test is more likely to serve as a barrier to women setting the lower-level goal of scheduling their first Pap test rather than the higher-level goal of maintaining yearly Pap screening. This finding underscores the importance of designing interventions that specifically educate women who have never received a Pap test about the Pap test procedure and instruments used, as well as educating healthcare providers about women’s attitudes and beliefs regarding the Pap test, and useful communication techniques to allay women’s discomfort during a Pap test.' (p. 373) | 4 |
| Gele et al. (2017) | Barriers and facilitators to cervical cancer screening among Pakistani and Somali immigrant women in Oslo: a qualitative study | Focus groups | Migration background/ethnic minority | 25-70 | 35 | NO | Cervical cancer screening | The results show individual, sociocultural and system-related barriers, in addition to a facilitator for cervical cancer screenings among women.' (p. 493) | 4 |
| Ghebre et al. (2015) | Cervical Cancer: Barriers to Screening in the Somali Community in Minnesota | Interviews | Migration background/ethnic minority | 18+ | 23 | USA | Cervical cancer screening | 'Informant interviews conducted among Somali women in Minnesota revealed multiple barriers to cervical cancer screening. Using the socio-ecological framework to group the barriers, we were able to categorize them into three major levels, to make sense of which barriers overlap and at what levels.' (p. 726) | 5 |
| Ghebrendrias et al. (2021) | An Examination of Misconceptions and Their Impact on Cervical Cancer Prevention Practices among Sub-Saharan African and Middle Eastern Refugees | Focus groups | Migration background/ethnic minority | 20-50 | 53 | USA | Cervical cancer screening | 'This study examined the cervical cancer prevention practices of refugee women from the Middle East and SubSaharan Africa living in San Diego, California. Focus groups revealed shared patterns of avoidance of pap tests due to a fear of pelvic examinations and a concern for modesty and lack of knowledge regarding the HPV vaccine. Participants requested culturally centered health education through print and online platforms. From this pool of knowledge, providers can begin to grasp the extent of the disparity and target future efforts toward a multimodal approach to cervical cancer prevention.' (p. 388) | 5 |
| Gorman & Porteous (2018) | Influences on Polish migrants' breast screening uptake in Lothian, Scotland | Interviews | Migration background/ethnic minority | mean: 58 | 11 | UK | Breast cancer screening | 'We show that Polish women living in Scotland have diffi- culties accessing the breast screening programme. They currently get information from the SBSP primarily designed for Scottish women which does not address the specific concerns of Polish women. These concerns relate to Polish health culture and the Polish screening programme. We demonstrate that the information needs of women who come from a developed country such as Poland with existing screening programmes are different to those of women from the developing world with no screening programmes. ' (p. 90) | 4 |
| Grandahl et al. (2015) | Immigrant women's experiences and views on the prevention of cervical cancer: a qualitative study | Focus groups | Migration background/ethnic minority | 18-54 | 50 | SE | Cervical cancer screening | 'The women were positive to the prevention of cervical cancer and appreciated being called for check-ups, but had difficulty understanding the letter of invitation and communicating with health-care professionals. ' (p. 352) | 5 |
| Groenenberg et al. (2015) | 'Check it out!' Decision-making of people in vulnerable situations about participation in a two-stage cardiometabolic health check: a qualitative study | Focus groups | Migration background/ethnic minority and low SES | 45-70 | 119 | NL | Cardiometabolic screening | 'The purpose of this study was to provide an overview of informational, practical, and psychosocial factors influencing the (non-)participation in a two-stage cardiometabolic health check among difficult-to-reach, vulnerable populations. Even though similarities between determinants influencing (non-)participation in the HRA and the PCs were manifold, important differences were also noted. When considering filling out the HRA, more cognitive aspects, including rational cost–benefit considerations, were prominent. After a high-risk HRA result and the subsequent decision to (not) participate in the PCs, cognitive aspects would still play a role but more importantly would trigger negative affective responses and related coping aspects to deal with these emotions.' (p. 241) | 3 |
| Hamdiui et al. (2018) | Hepatitis B in Moroccan-Dutch: a qualitative study into determinants of screening participation | Interviews | Migration background/ethnic minority | Mean: 47 | 9 | NL | Hepatitis screening | 'This study identified important facilitators and barriers, which require careful consideration when designing and implementing communication strategies. Specific and accurate knowledge provision is important, but should be accompanied by strategies to address shame and stigma. Islamic religious leaders within the MoroccanDutch community should, therefore, be informed about hepatitis B and risk (i) to bring information across, (ii) to decrease elements of shame and stigma, leading to more acceptance of HBV screening.' (p. 921) | 3 |
| Ilangovan et al. (2016) | Acceptability and Feasibility of Human Papilloma Virus Self-Sampling for Cervical Cancer Screening | Surveys | Migration background/ethnic minority and low-income | Mean: 52 | 180 | USA | Cervical cancer screening | 'In these two safety-net sites, HPV self-sampling was feasible and had high acceptability among patients and staff.' (p. 949) | 2 |
| Jin et al. (2021) | Analyzing factors associated with decisional stage of adopting breast cancer screening among Korean American women using precaution adoption process model | Surveys | Migration background/ethnic minority | Mean: 58,1 | 281 | USA | Breast cancer screening | 'This study found suboptimal mammography screening outcomes in Korean American women and BC screening-related knowledge, decisional balance, and regular medical check-up, respectively, to be a factor associated with higher stage of adoption for BC screening.' (p. 443) | 4 |
| Jung et al. (2018) | The Chinese and Korean American immigrant experience: a mixed-methods examination of facilitators and barriers of colorectal cancer screening | Mixed | Migration background/ethnic minority | 50-85 | 137 | USA | Colorectal cancer screening | ' Future interventions and programs aiming to increase CRC screening among Chinese and Korean Americans should address both cultural and non-cultural factors that influence CRC screening uptake.' (p. 847) | 4 |
| Kawar (2013) | Barriers to breast cancer screening participation among Jordanian and Palestinian American women | Interviews | Migration background/ethnic minority | Mean: 46,7 | 107 | USA | Breast cancer screening | ' Clinicians should be cognizant of the culture, beliefs and practices of Arab Middle Eastern immigrant women and the influence of these factors on their decision to participate in routine BCS. To increase participation in BCS and knowledge of breast cancer, appropriate language and culturally sensitive educational materials should be created and made available to Arab Middle Eastern immigrant women.' (p. 88) | 2 |
| Kenya et al. (2015) | Human Papilloma Virus and Cervical Cancer Education Needs among HIV-Positive Haitian Women in Miami | Focus groups | Migration background/ethnic minority & living with (other) illness | 30-60 | 21 | USA | Cervical cancer screening | 'There is critical need for culturally relevant interventions to improve HPV/cervical cancer knowledge among HIV-positive Haitian women.' (p. 262) | 2 |
| Kerkhoff et al. (2022) | Integrating Rapid Diabetes Screening Into a Latinx Focused Community-Based Low-Barrier COVID-19 Testing Program | Surveys | Migration background/ethnic minority | 29-51 | 6631 | USA | Diabetes screening | 'In this study, we found that a strategy to make point-of-care diabetes testing available to low-income Latinx community members seeking COVID-19 testing at a community site in the Mission District in San Francisco was feasible to implement, highly acceptable to individuals tested, and reached our priority population of low-income Latinx persons not engaged in formal health care services.' (p. 9) | 2 |
| Kim et al. (2018) | Decisional balance and self-efficacy mediate the association among provider advice, health literacy and cervical cancer screening | Surveys | Migration background/ethnic minority | 21-65 | 560 | USA | Cervical cancer screening | 'Findings from this study suggest possible pathways through which provider advice and health literacy affect Pap tests. Interventions targeting immigrant women with limited English proficiency should consider skill-based approaches such as health literacy training, promoting patient-provider communications and emphasizing decisional balance and self-efficacy as potentially sustainable ways of promoting Pap tests.' (p. 55) | 2 |
| Lee et al. (2014) | Barriers to cancer screening among Medical Aid Program recipients in the Republic of Korea: a qualitative study | Focus groups | Low SES | 35-79 | 23 | Republic of Korea | Cervical, gastric and liver cancer screening | We identified six barriers to screening: lack of trust in the NCSP and cancer screening units; fear of being diagnosed with cancer; discomfort or pain from the screening procedure; lack of time, lack of knowledge about cancer screening or lack of awareness of the existence of the NCSP; physical disability or underlying disease; and logistic barriers.' (p. 1) | 3 |
| Lee et al. (2016) | Factors associated with receiving pap tests among married immigrant women of Vietnamese origin in southern Taiwan | Surveys | Migration background/ethnic minority | Mean: 35,55 | 427 | TW | Cervical cancer screening | 'This study found that having children, knowledge of Pap tests, and barriers to receiving Pap tests were all behaviors related to receiving Pap tests. Information and strategies that are culturally adapted to Taiwan should be developed to provide Vietnamese–Chinese bilingual documents, encouraging Vietnamese women in transnational marriages to discuss Pap tests and related issues with family members, promoting Pap tests as part of routine health education topics, providing a mobile service for women who cannot schedule Pap tests because of work or family duties, referring women to female physicians for Pap tests to reduce embarrassment, and strengthening health education programs specific to Vietnamese women in transnational marriages who do not have children.' (pp. 253-254) | 2 |
| Lee & Im (2013) | Colorectal Cancer Screening among Korean American Immigrants: Unraveling the Influence of Culture | Surveys | Migration background/ethnic minority | 50-88 | 281 | USA | Colorectal cancer screening | 'Binary logistic regression analyses revealed significant predictors including health belief constructs, such as perceived seriousness of cancer and confidence in screening uptake, and gender- specifi c cultural beliefs and attitudes about CRC screening. Perceived helplessness lowered CRC screening among the women, while fatalism lowered it among the men. The findings reinforce a need for cultural- and gender- specific intervention strategies to increase CRC screening in this particularly vulnerable population.' (p. 579) | 3 |
| Lee & Jung (2013) | Older Korean American Men's Prostate Cancer Screening Behavior: The Prime Role of Culture | Surveys | Migration background/ethnic minority | Mean: 67,03 | 134 | USA | Prostate cancer screening | 'Findings from our study confirmed the importance of culture-based beliefs about cancer and health care on participants’ decisions about preventative measures. A crisisoriented intervention approach to cancer and physical modesty were found to be associated with a substantially reduced likelihood of recent prostate cancer screening.' (p. 1034) | 2 |
| Lee & Lee (2017) | Barriers to Cervical Cancer Screening and Prevention in Young Korean Immigrant Women: Implications for Intervention Development | Focus groups | Migration background/ethnic minority | 21-29 | 16 | USA | Cervical cancer screening | 'The current study examined barriers to Pap test uptake and HPV vaccination among young Korean immigrant women. Findings suggested that the focus group participants have limited knowledge of cervical cancer and preventive behaviors' (pp. 357-358) | 4 |
| Lee & Lee (2013) | Korean Americans' Beliefs about Colorectal Cancer Screening | Interviews | Migration background/ethnic minority | 50+ | 26 | USA | Colorectal cancer screening | 'Results show the critical need for in-depth understanding of unique health and cultural beliefs about CRC screening in KAs. These beliefs could be useful for future intervention strategies to change health and cultural beliefs in order to increase CRC screening participation in KAs.' (p. 45) | 5 |
| Leng (2022) | Chinese Livery Drivers' Perspectives on Adapting a Community Health Worker Intervention to Facilitate Lung Cancer Screening | Focus groups | Migration background/ethnic minority & nonstandard work arrangements | 21-80 | 39 | USA | Lung cancer screening | 'Focus group participants were uncertain about whether smoking was associated with cancer, unfamiliar with LCS, and reported numerous barriers to LCS uptake. Most believed a CHW program to facilitate LCS would be acceptable and feasible, if tailored to meet their needs. Our results have implications for improving access to early detection of lung cancer and preventive care (e.g., culturally appropriate smoking cessation and health education programs) for Chinese livery drivers.' (p. 332) | 4 |
| Lin et al. (2016) | Barriers of Female Breast, Colorectal, and Cervical Cancer Screening Among American Indians-Where to Intervene? | Surveys | Migration background/ethnic minority | 21+ | 199 | USA | Colorectal, breast and cervical cancer screening | 'The study found that knowledge about cancer screening, geographic access to PCPs (e.g., distance and transportation), as well as socioeconomic factors were significant barriers for breast, cervical, and colorectal cancer screening. Based on these findings, the study integrated geographic access and socioeconomic factors to identify geographic areas where people had low access to cancer screening in South Dakota.' (p. 904) | 3 |
| Lofters et al. (2021) | Care in the Community: Opportunities to improve cancer screening uptake for people living with low income | Focus groups and interviews | Low-income | 32-71 | 28 | CA | Colorectal, breast and cervical cancer screening | 'Primary care patients living with low income and who were or had recently been overdue for cancer screening recommended taking a community outreach approach in order to reduce socioeconomic disparities in cancer screening uptake.' (p. 4) | 2 |
| Lofters et al. (2020) | A "Tea and Cookies" Approach: Co-designing Cancer Screening Interventions with Patients Living with Low Income | Focus groups and interviews | Low-income | 32-71 | 28 | CA | Colorectal, breast and cervical cancer screening | 'We used input from patients living with low income to co-design a new approach to cancer screening in our primary care organization, an approach that could be broadly applicable to other contexts and settings. We learned from our patients that a multi-modal strategy will likely be best to maximize screening uptake.' (p. 255) | 5 |
| Luque et al. (2018) | Utilization of Cervical Cancer Screening Among Hispanic Immigrant Women in Coastal South Carolina | Surveys | Migration background/ethnic minority | 21-64 | 196 | USA | Cervical cancer screening | 'Differences in cervical cancer screening for participants were partially explained by psychosocial factors, health status, and individual and structural barriers to healthcare.' (p. 589) | 3 |
| Madhivanan et al. (2016) | Family and cultural influences on cervical cancer screening among immigrant Latinas in Miami-Dade County, USA | Focus groups | Migration background/ethnic minority | Mean: 35,4 | 35 | USA | Cervical cancer screening | 'The findings of this study suggest that Latina perceptions about the role of family in cervical cancer screening are far more diverse than the current literature suggests. They also highlight the role of country of origin in shaping some of the beliefs and misconceptions that guide cervical cancer screening behaviour. While the family appears to still be an important factor in many women’s health behaviour, there is also evidence that an increasing number of women are taking decisions themselves and using family in less traditional ways such as motivators and sources of current information rather than arbiters of decision making' (p. 719) | 4 |
| Malika et al. (2020) | African Immigrant Health: Prostate Cancer Attitudes, Perceptions, and Barriers | Focus groups and interviews | Migration background/ethnic minority | 20+ | 33 | USA | Prostate cancer screening | 'The results of this qualitative study unveiled perceptions, attitudes, beliefs, and knowledge of PCa among African immigrants that should inform the planning, development, and implementation of preventive programs to promote men’s health and PCa awareness.' (p. 1) | 4 |
| Manne et al. (2015) | Colorectal Cancer Screening Among Foreign-born South Asians in the Metropolitan New York/New Jersey Region | Surveys | Migration background/ethnic minority | 50-75 | 208 | USA | Colorectal cancer screening | 'In conclusion, awareness of CRC screening and uptake of screening was low in this population of foreign-born South Asians. Interventions to promote CRC screening may benefit from targeting this subgroup of Asian Americans.' (p. 1075) | 3 |
| Manne et al. (2021) | Culturally-adapted behavioral intervention to improve colorectal cancer screening uptake among foreign-born South Asians in New Jersey: the Desi Sehat trial | Surveys | Migration background/ethnic minority | 50,7-75 | 93 | USA | Colorectal cancer screening | 'Desi Sehat was a well-evaluated and participation in the session was high, participant knowledge significantly increased, and screening barriers, worry about CRC, and worry about CRC screening tests declined significantly. Future studies should focus on enhancing recruitment and retention and include a randomized control design.' (p. 554) | 3 |
| March et al. (2018) | Barriers to and discourses about breast cancer prevention among immigrant women in Spain: a qualitative study | Interviews | Migration background/ethnic minority and low SES | 50-69 | 36 | ES | Breast cancer screening | 'The culture of origin affects whether an immigrant has a fatalistic or proactive approach toward breast cancer screening. Immigrants from low-income countries and Spanish natives from a low socioeconomic class experience barriers in access to breast cancer screening. Frequently changing homes is also a barrier for immigrant women.' (p. 1) | 4 |
| Marques et al. (2023) | What is the role of attitudinal barriers on cervical cancer screening non-attendance? Findings from a cross-sectional study with migrant women in Portugal | Surveys | Migration background/ethnic minority | 20+ | 1100 | PT | Cervical cancer screening | 'Based on the fndings, out of all the factors analyzed, low perceived need of screening and lack of motivation are the most relevant factors associated with non-attendance among migrants in Portugal. Promoting health literacy and empowering women with knowledge about benefts of screening may help overcoming these barriers.' (p. 1) | 3 |
| Montealegre et al. (2015) | Feasibility of Cervical Cancer Screening Utilizing Self-sample Human Papillomavirus Testing Among Mexican Immigrant Women in Harris County, Texas: A Pilot Study | Surveys | Migration background/ethnic minority | Median: 38 | 100 | USA | Cervical cancer screening | 'In conclusion, self-sample HPV testing is highly acceptable to Mexican immigrant screening non-attendees. Its high acceptability and low cost make it a promising strategy for identifying HR–HPV positive women who are otherwise missed by existing cytology-based cervical cancer screening programs. However, more needs to be done to address critical barriers to healthcare utilization that limit HR–HPV positive women’s ability to attend for clinical follow-up. Without such follow-up, self-sample HPV testing is of little or no value.' (p. 710) | 2 |
| Nguyen-Truong et al. (2018) | Vietnamese American Women's Beliefs and Perceptions About Breast Cancer and Breast Cancer Screening: A Community-Based Participatory Study | Focus groups | Migration background/ethnic minority | Mean: 46,45 | 40 | USA | Breast cancer screening | 'This study provides valuable insights for practice. HCPs have a crucial role in promoting positive health behaviors and providing culturally sensitive interventions to increase BC screening' (p. 561) | 3 |
| Nguyen-Truong et al. (2017) | Feasibility of a Multicomponent Breast Health Education Intervention for Vietnamese American Immigrant Women | Surveys | Migration background/ethnic minority | 50-80 | 40 | USA | Breast cancer screening | 'The recruitment response rate was 58%. Knowledge about breast cancer, breast cancer susceptibility, and the benefits of mammography as related to breast cancer significantly increased following the intervention. Acceptability of the targeted program, good feasibility, and very low attrition was achieved.' (p. 615) | 2 |
| Nolan et al. (2014) | Barriers to Cervical Cancer Screening and Follow-up Care among Black Women in Massachusetts | Focus groups | Migration background/ethnic minority | 35+ | 17 | USA | Cervical cancer screening | 'Findings from interviews revealed that inadequate information and education of providers and patients create barriers to appropriate screening and treatment practices for Black women. Fear, cultural beliefs, and compounding factors related to poverty, gender roles, and health system barriers create delays to screening and follow-up care. Also, unconscious bias, therapeutic delays, and miscommunication are important factors affecting continuity of care. These results suggest a need for comprehensive, culturally specific cervical cancer prevention education initiatives and interventions for Black women and strategies to improve patient–provider relationships.' (p. 580) | 4 |
| Palmer et al. (2014) | Reasons for non-uptake and subsequent participation in the NHS Bowel Cancer Screening Programme: a qualitative study | Focus groups | Migration background/ethnic minority and IMD quintiles | N/A | 243 | UK | Colorectal cancer screening | ' Initiatives to normalise discussion about bowel cancer screening, to link the BCSP to general practice, and to simplify the test itself may lead to increased uptake across all social groups.' (p. 1705) | 5 |
| Patel et al. (2014) | Factors influencing breast cancer screening in low-income African Americans in Tennessee | Surveys | Migration background/ethnic minority | 40+ | 334 | USA | Breast cancer screening | 'There were several predictors of breast cancer screening such as marital status and having health insurance (P<0.05). Additionally, there were associations between obstacles to screening and geographic region such as transportation and not having enough information about screenings (P< .05). Educational interventions aimed at improving breast cancer knowledge and screening rates should incorporate information about obstacles and predictors to screening.' (p. 943) | 4 |
| Pratt et al. (2020) | Testing a Religiously Tailored Intervention with Somali American Muslim Women and Somali American Imams to Increase Participation in Breast and Cervical Cancer Screening | Surveys | Migration background/ethnic minority | Mean: 47,3 | 30 | USA | Breast and cervical cancer screening | 'Religiously tailored messages can be an important community asset that can be used to support eforts to engage Somali American Muslim women in the value of breast and cervical cancer screening. Group-level community leaders, such as imams, can also be positively engaged in those eforts. Drawing on religiously tailored approaches in cancer screening eforts could ofer more culturally relevant and meaningful content for Somali American Muslim patients and help address the cancer disparities facing the community.' (p. 93) | 4 |
| Pratt et al. (2017) | Views of Somali women and men on the use of faith-based messages promoting breast and cervical cancer screening for Somali women: a focus-group study | Focus groups | Migration background/ethnic minority | N/A | 34 | USA | Breast and cervical cancer screening | 'Somali immigrant women and men found faith-based messages addressing topics of predestination and modesty and encouraging the use of screening and treatment to be both acceptable and influential. Faith can play an important role as an asset to promote breast and cervical cancer screening, and there may be substantial benefits to adding faith-based messaging to other interventions that focus on improving screening uptake.' (p. 1) | 4 |
| Racine et al. (2022) | Examination of Breast Cancer Screening Knowledge, Attitudes, and Beliefs among Syrian Refugee Women in a Western Canadian Province | Surveys | Migration background/ethnic minority | Mean: 37,9 | 75 | CA | Breast cancer screening | 'Participants’ breast cancer screening practices were low. Health beliefs, Arab culture and stigma about cancer affected women’s BCS practices. Faith-based interventions may improve knowledge and practices.' (p. 177) | 3 |
| Ramos et al. (2016) | Perspectives on breast health education and services among | Focus groups | Migration background/ethnic minority | Average: 42 | 37 | USA | Breast cancer screening | Health is more than just clinical care, and therefore, it is important to understand the contextual and cultural factors that have resulted in low screening rates and develop methods to address these them. Failure to address these aspects of social determinants of health could hamper efforts to improve breast health and reduce disparities. | 4 |
| Raymond et al. (2014) | Culturally informed views on cancer screening: a qualitative research study of the differences between older and younger Somali immigrant women | Focus groups | Migration background/ethnic minority | 20-65 | 29 | USA | Breast and cervical cancer screening | 'Cultural misperceptions and attitudes need to be addressed in developing culturally-appropriate interventions to improve screening uptake for Somali women. A nuanced response is required to address barriers specific to younger and older groups. Culturally informed beliefs can be integrated into intervention development, preventive care and screening promotion.' (p. 1) | 5 |
| Raynault et al. (2020) | Social inequalities in breast cancer screening: evaluating written communications with immigrant Haitian women in Montreal | Focus groups and interviews | Migration background/ethnic minority and deprived areas | 40-69 | 32 | CA | Breast cancer screening | 'The main conclusion is that the technical wording of the letter did not match participants’ literacy levels, which created a major obstacle to adherence to the program.' (p. 8) | 4 |
| Ridgeway et al. (2021) | Closing the gap: Participatory formative evaluation to reduce cancer screening disparities among patients with limited English proficiency | Interviews | Migration background/ethnic minority | 50-74 | 9 | USA | Colorectal, breast and cervical cancer screening | Findings from this study indicate that it is possible to engage a diverse group of stakeholders in strategies that are responsive to health care providers and patients, including LEP patients from heterogeneous backgrounds. | 3 |
| Rogers et al. (2021) | A qualitative study of barriers and enablers associated with colorectal cancer screening among Somali men in Minnesota | Interviews | Migration background/ethnic minority | 50-74 | 27 | USA | Colorectal Cancer screening | 'The low rate of CRC screening among under-resourced minority men, especially those of Somali origin, is a continuing challenge to public health. To develop effective interventions that may change preventive behavior, it is critical to identify the beliefs, attitudes, intentions, and other factors influencing members of this group.' (p. 15) | 3 |
| Saadi et al. (2015) | Bosnian, Iraqi, and Somali Refugee Women Speak: A Comparative Qualitative Study of Refugee Health Beliefs on Preventive Health and Breast Cancer Screening | Interviews | Migration background/ethnic minority | 23-75 | 57 | USA | Breast cancer screening | 'Taken together, duration of time in United States and prior exposure to Western medicine account for differences in refugee women’s knowledge of preventive care. Understanding population-specific health beliefs, health information, and behavior are crucial for designing tailored prevention programs for refugee women.' (p. 501) | 3 |
| Sage et al. (2020) | Girl, just pray ...: Factors That Influence Breast and Cervical Cancer Screening Among Black Women in Rochester, MN | Mixed | Migration background/ethnic minority | Average: 36,5 | 45 | USA | Cervical cancer screening | 'Black women face real and perceived barriers to cancer screening even where health resources are abundant. Results reiterate an on going need for culturally appropriate interventions to improve Black women’s breast and cervical cancer screening participation by minimizing barriers and engaging entire communities - including Black women, religious leaders, and HCPs.' (p. 454) | 3 |
| Salad et al. (2015) | A Somali girl is Muslim and does not have premarital sex. Is vaccination really necessary? A qualitative study into the perceptions of Somali women in the Netherlands about the prevention of cervical cancer | Focus groups and interviews | Migration background/ethnic minority | 23-66 | 32 | NL | Cervical cancer screening | 'Current measures in the Netherlands to prevent women from developing cervical cancer hardly reach Somali women because these women perceive these kinds of preventative measures as not personally relevant. Dutch education strategies about cervical cancer deviate from ways of exchanging information within the Somali community. Teachers can provide culturally sensitive information to young Somali women in schools. For Somali mothers, oral education (e.g., poetry or theater) about the Dutch health care system and men’s roles in HPV transmission may be useful.' (p. 1) | 3 |
| Schoenberg et al. (2013) | Appalachian women's perspectives on breast and cervical cancer screening | Focus groups and interviews | Migration background/ethnic minority and low SES | 18-71 | 79 | USA | Breast and cervical cancer screening | 'The updated and novel insights provided by community members and interpreted in terms of consumer information processing theory emphasize the key role that healthcare providers play in increasing early cancer detection efforts. Greater attention to these recommendations may help reduce the deadly cancer inequities in the Appalachian region of the USA, as well as in other traditionally underserved regions globally.' (p. 11) | 3 |
| Schuster et al. (2018) | They were just waiting to die: Somali Bantu and Karen Experiences with Cancer Screening Pre- and Post-Resettlement in Buffalo, NY | Focus groups and interviews | Migration background/ethnic minority | Mean: 32,5 and 36,6 | 30 | USA | Cancer screening | 'There is a need for cancer education among the Somali Bantu and Karen refugee communities in Buffalo, NY that incorporates a life course perspective in order to recognize the limited experience with preventive health care services and pre-resettlement exposure to cancer.' (p. 243) | 4 |
| Seay et al. (2015) | Cancer Screening Utilization Among Immigrant Women in Miami, Florida | Surveys | Migration background/ethnic minority | 30+ | 234 | USA | Colorectal, breast and cervical cancer screening | 'Given the disparities in cancer screening between our samples and the larger sociodemographic groups in which they are often included, targeted approaches that address structural barriers (lack of health insurance or usual source of care) may improve access to cancer screening among recent immigrants. Community partnerships may be essential in facilitating the interventions needed to overcome cancer-related disparities in these groups.' (p. 11) | 4 |
| Seibert et al. (2017) | National Disparities in Colorectal Cancer Screening Among Obese Adults | Surveys | Obese (underscreened subpopulation) | 50-75 | 8550 | USA | Colorectal cancer screening | 'In line with the updated USPSTF screening recommendations, healthcare providers need to recognize the unique screening barriers of obese men and women and individualize testing strategies to encourage adherence; doing so will promote screening uptake and may lessen disparities among the vulnerable populations most affected by obesity.' (p. 48) | 4 |
| Sentell et al. (2013) | Colorectal cancer screening: low health literacy and limited English proficiency among Asians and Whites in California | Surveys | Low-literare | 50-75 | 15888 | USA | Colorectal cancer screening | 'This study confirms that health literacy is associated with CRC screening in a large population-based sample. We also provide insight to significant health communication predictors for CRC screening among Asian Americans generally, a group with notable cancer screening and mortality disparities. Unlike race/ethnicity and many other factors associated with cancer disparities, health literacy is amenable to direct intervention at the individual, patient/provider, and systems levels and may be a fruitful focus for intervention.' (pp. 251-252) | 4 |
| Seo et al. (2018) | Cervical Cancer Screening Experiences Among Chinese American Immigrant Women in the United States | Interviews | Migration background/ethnic minority | 34-64 | 12 | USA | Cervical cancer screening | 'Women’s HCPs should be aware of and give consideration to cultural differences through the provision of more educational information and comfort to Chinese immigrant women who seek cervical cancer screening. Ultimately, the development of culturally appropriate and affordable cancer prevention programs with effective strategies is important to ease Chinese American women’s senses of vulnerability.' (p. 52-53) | 4 |
| Shirazi et al. (2013) | Afghan immigrant women's knowledge and behaviors around breast cancer screening | Interviews | Migration background/ethnic minority | Mean: 46 | 53 | USA | Breast cancer screening | 'Findings showed low levels of knowledge and awareness about breast cancer and low utilization of early-detection examinations for breast cancer among participants. The findings also suggest a significant need for a community-based breast health education program that recognizes the unique social, cultural, and religious dynamics of the Muslim Afghan community.' (p. 1705) | 3 |
| Shokar et al. (2022) | Mediators of screening uptake in a colorectal cancer screening intervention among Hispanics | Surveys | Migration background/ethnic minority | Mean: 56 | 723 | USA | Colorectal cancer screening | 'This study contributes to our understanding of mediators of CRC screening and suggests that targeting self-efcacy, perceived benefts and fatalism could maximize the efectiveness of CRC screening interventions particularly in Hispanic populations.' (p. 1) | 4 |
| Silko (2017) | Increasing Breast Cancer Screening in Russian Immigrant Women: Identifying Barriers and Providing On-Site Mammography | Mixed | Migration background/ethnic minority | 40-62 | 27 | USA | Breast cancer screening | 'Providing access to on-site mammography has been shown to be an effective tool to reach communities that otherwise would not have access to these screenings.' (p. 71) | 2 |
| Simon et al. (2017) | Breast Cancer Screening Beliefs Among Older Chinese Women in Chicago's Chinatown | Focus groups | Migration background/ethnic minority | 45+ | 47 | USA | Breast cancer screening | 'This qualitative study highlights the increased need to connect Chinese older adults with resources and assistance to navigate the health care system. Many Chinese older adults are disadvantaged in the United States due to cultural, linguistic, and structural barriers and are vulnerable to poor health and low cancer screening utilization. Nevertheless, these challenges represent tremendous opportunities for community stakeholders, researchers, health professionals, social service agencies, and policy makers to improve the health and well-being of Chinese Americans.' (p. 39) | 5 |
| Sin et al. (2016) | Sociocultural Barriers to Lung Cancer Screening Among Korean Immigrant Men | Focus groups and interviews | Migration background/ethnic minority | Mean: 69 | 24 | USA | Lung cancer screening | 'While health care provider recommendations are important to Korean men’s uptake of screening procedures, few Korean men who are eligible for lung cancer screening have received a health care provider recommendation for the test.' (p. 796) | 4 |
| Soto et al. (2018) | Preferences of Underserved Chilean Women on a Mobile Technology Intervention for Cervical Cancer Screening: Qualitative Study | Focus groups | From deprived areas | 25-64 | 27 | CL | Cervical cancer screening | 'This study describes the opinions of women nonadherent to Pap testing on the potential use of mobile technologies for cervical cancer screening. Although the overall acceptance was positive, older women prefer personal contact and phone calls over text messaging.' (pp. 1-2) | 3 |
| Tatari et al. (2021) | The SWIM study: Ethnic minority women's ideas and preferences for a tailored intervention to promote national cancer screening programmes - A qualitative interview study | Focus groups and interviews | Migration background/ethnic minority and from deprived areas | 27-59 | 37 | DK | Colorectal, breast and cervical cancer screening | 'Ethnic minority women were interested in a tailored intervention, and they were keen to contribute with ideas and preferences. The findings emphasized the potential of a tailored intervention with specific suggestions to the context when attempting to reduce inequality in cancer screening participation.' (p. 1692) | 5 |
| Tatari (2020) | Perceptions about cancer and barriers towards cancer screening among ethnic minority women in a deprived area in Denmark - a qualitative study | Focus groups and interviews | Migration background/ethnic minority and from deprived areas | 27-59 | 37 | DK | Colorectal, breast and cervical cancer screening | 'Ethnic minority women did not have sufficient knowledge about cancer and the purpose of cancer screening. Perceptions about cancer screening were characterised by openness and the study showed positive and curious attitudes towards screening participation. The findings emphasise the importance of culturally adapted interventions for ethnic minority women in attempts to reduce inequality in screening participation.' (p. 1) | 3 |
| Team et al. (2013) | From state care to self-care: cancer screening behaviours among Russian-speaking Australian women | Interviews | Migration background/ethnic minority and caregiver | Mean: 62 | 8 | AU | Breast and cervical cancer screening | 'We argue therefore that GPs’reminders of screening and referrals to Pap testing and mammography may increase Russian-speaking women’s access to reproductive screening and ultimately improve their health outcomes. Australian health care policy related to reproductive health screening may need to be modified with regard to reminders. National screening registers send reminders to women who have not presented for routine screening. However, our study indicates that Russian-speaking immigrant women prefer to receive personal reminders sent to them from their own doctors, and prompts during the face-to-face consultation to present for screening; anonymous automated reminders sent by registers are less likely to be effective.' (p. 135) | 3 |
| Umaefulam et al. (2023) | The co-development of a linguistic and culturally tailored tele-retinopathy screening intervention for immigrants living with diabetes from China and African-Caribbean countries in Ottawa, Canada | Focus groups | Migration background/ethnic minority and living with illness | 18+ | 13 | CA | Diabetes screening | 'We highlight the co-development of a linguistically and culturally tailored tele-retinopathy intervention with patient and health system partners to improve the attendance of DRS for immigrants to Canada from China and African-Caribbean countries. ' (p. 18) | 4 |
| Vahabi & Lofters (2016) | Muslim immigrant women's views on cervical cancer screening and HPV self-sampling in Ontario, Canada | Focus groups | Migration background/ethnic minority | 21-69 | 30 | CA | Cervical cancer screening | 'To enhance Muslim immigrant women screening uptake, efforts should made to increase 1) their knowledge of the Canadian health care system and preventive services at the time of entry to Canada, and 2) access to culturally sensitive education programs, female health professionals, and alternative modes of screening like HPV self-sampling. Health professionals need to take an active role in offering screening during health encounters, be educated about sexual health communication with minority women, and be aware of the detrimental impact of preconceived assumptions about sexual activity of Muslim women.' (p. 1) | 5 |
| van Allen et al. (2021) | Barriers to and enablers of attendance at diabetic retinopathy screening experienced by immigrants to Canada from multiple cultural and linguistic minority groups | Interviews | Migration background/ethnic minority and living with illness | Mean: 47,49 | 39 | CA | Diabetes screening | 'Our results can inform linguistic and culturally competent interventions to support immigrants living with diabetes in attending eye screening to prevent avoidable blindness.' (p. 2) | 5 |
| Van Hemelrijck et al. (2019) | Breast cancer screening and migrants: exploring targeted messages for Moroccan migrant women in Brussels | Focus groups | Migration background/ethnic minority | Mean: 45 | 32 | BE | Breast cancer screening | 'Alternative packages were appreciated by some, but a number of adjustments did not catch participants’ attention. Printed communication delivered by post does not appear to be the preferred means of communication to encourage breast cancer screening for Moroccan migrant women in Brussels, nor does it seem appropriate to address the barriers to screening found in this study. The benefit of targeted postal invitation packages for Moroccan migrant women in Brussels seems limited for a variety of reasons.' (p. 927) | 5 |
| Weinstein et al. (2019) | Feasibility Pilot Outcomes of a Mammography Decision Support and Navigation Intervention for Women With Serious Mental Illness Living in Supportive Housing Settings | Mixed | Living with (other) illness | Mean: 53,19 | 21 | USA | Breast cancer screening | 'The purpose of this study was to examine the outcomes, feasibility, and acceptability of a mammography decision support and navigation intervention women with SMI living in supportive housing settings. We partially confirmed our primary hypotheses. While there were no significant changes in knowledge, attitudes, or intention to screen (likely because of high baseline levels of knowledge and orientation toward screening), there was a statistically significant decrease in decisional conflict.' (p. 7) | 3 |
| Woudstra et al. (2015) | Knowledge, attitudes and beliefs regarding colorectal cancer screening among ethnic minority groups in the Netherlands - a qualitative study | Interviews | Migration background/ethnic minority | 48-74 | 30 | NL | Colorectal cancer screening | 'To ensure that all eligible individuals, including ethnic minority groups, have equal opportunities to informed participation in screening, targeted communication strategies should be developed, such as oral and visual channels, and face-to-face communication in the mother tongue. This will help ethnic minority groups to make an informed decision about participation in CRC screening.' (p. 1312) | 5 |
| Zehbe et al. (2017) | Self-administered versus provider-directed sampling in the Anishinaabek Cervical Cancer Screening Study (ACCSS): a qualitative investigation with Canadian First Nations women | Focus groups | Migration background/ethnic minority | 18-70 | 69 | CA | Cervical cancer screening | 'We show that self-sampling in conjunction with community engagement and culturally sensitive education and could be a viable option for underscreened Canadian First Nations women.' (p. 1) | 2 |
| Zhao et al. (2013) | Hepatitis B knowledge and preventive practices of Chinese American immigrants in Southern California | Surveys | Migration background/ethnic minority | Mean: 44 | 179 | USA | Hepatitis screening | 'Most participants were knowledgeable of HBV (91.6%) or HBV vaccination (70.5%), yet screening and vaccination for the virus were low (36.9% and 26.3%, respectively). Low knowledge was also found regarding modes of disease transmission. Barriers to engage preventive practices included: “feeling well/no health problems” and “not suggested by a doctor.” These findings suggest the need for further education.' (p. 205) | 3 |

1. We assessed the methodological quality of each of the included studies through the use of the Mixed Methods Appraisal Tool (MMAT) version 2018. Quality score ranged from 0 to 5, where 0 means that none of the five criteria were met and 5 means that all criteria were met. [↑](#footnote-ref-1)
