# Supplementary material for: Who is left out? A systematic review on the barriers and facilitators for screening participation reported by people in vulnerable situations with strategies for the future
Source: Public Health Pract (Oxf). 2026 May 8;11:100803. doi: 10.1016/j.puhip.2026.100803 (PMC13194543; doi:10.1016/j.puhip.2026.100803)
Supplement: Multimedia component 2 [file mmc2.docx]

# Appendix A: Search strategy

| **Information source** | **Filters** | **Search fields** | **Concept** | **Search query** | |
| --- | --- | --- | --- | --- | --- |
|  |  |  |  | **Free text** | **MeSH terms** |
| PubMed incl. Medline | English language AND year=2013-search date (June 2023) | All | 1: People in vulnerable situations | ("low* socioeconomic status" or "low* socio-economic status" or "low* SES" or "low* income level*" or "low* health literacy" or "low* literacy" or "socioeconomic* disadvantage*" or "socio-economic* disadvantage*" or "socioeconomic inequit*" or "socio-economic inequit*" or "socioeconomic inequalit*" or "socio-economic inequalit*" or "disadvantaged group*" or "disadvantaged communit*" or "deprived area*" or "deprived communit*" or "deprived neighborhood*" or "deprived neighbourhood*" or immigra* or "hard-to-reach population*" or "health inequit*" or "low* education" or "pover*").mp. or ("vulnerable populations").mp or (exp Vulnerable populations/) | "Low Socioeconomic Status"[Mesh]) OR "Healthcare Disparities"[Mesh] OR "Health Inequities"[Mesh] OR "Socioeconomic Factors"[Mesh] OR "Cultural Deprivation"[Mesh]OR "Emigrants and Immigrants"[Mesh] OR "Undocumented Immigrants"[Mesh] OR "Educational Status"[Mesh] OR "Poverty Areas"[Mesh] OR "Poverty"[Mesh] |
|  |  |  | 2: Screening | screening.mp. or exp Mass Screening/ or exp Mass Screening/ or screening programme.mp. or exp "Early Detection of Cancer"/ | "Mass Screening"[Mesh] OR "Early Detection of Cancer"[Mesh] OR "diagnosis" [Subheading] OR "Diagnostic Screening Programs"[Mesh] OR "Direct-To-Consumer Screening and Testing"[Mesh] |
|  |  |  | 3: (Non)participation | ("participat*" or "nonparticip*" or "non-participat*" or "utiliz*" or "utilis*" or "uptake" or "up-take" or "take-up" or "takeup").mp | "Patient Participation"[Mesh] OR "Community Participation"[Mesh] OR "Social Marginalization"[Mesh] OR "Refusal to Participate"[Mesh] OR "Delivery of Health Care"[Mesh] |
|  |  |  | 4: Barriers and facilitators | ("barrier*" or "obstacle*" or "facilitat*" or "enable*" or "motivat*" or "challeng*" or "resistan*").mp. | "Motivation" [Mesh] |
|  |  |  | 5: Chronic disease | chronic disease.mp. or exp Chronic Disease/ or exp Cardiovascular Diseases/ or exp Lung Diseases/ or Neoplasms/ or exp Diabetes Mellitus, Type 2/ or exp Renal Insufficiency, Chronic/ | "Chronic Disease"[Mesh] OR "Chronic Disease Indicators"[Mesh] OR "Noncommunicable Diseases"[Mesh] OR "Neoplasms"[Mesh] OR "Early Detection of Cancer"[Mesh] OR "Cardiovascular Diseases"[Mesh] OR "Lung Diseases, Obstructive"[Mesh] OR "Pulmonary Disease, Chronic Obstructive"[Mesh] OR "Chronic Kidney Diseases of Uncertain Etiology"[Mesh] OR "Renal Insufficiency, Chronic"[Mesh] OR "Public Health"[Mesh] OR "Public Health Surveillance"[Mesh] |
| PsycINFO | English language AND year=2013-search date (June 2023) | All | 1: People in vulnerable situations | ("low* socioeconomic status" or "low* socio-economic status" or "low* SES" or "low* income level*" or "low* health literacy" or "low* literacy" or "socioeconomic* disadvantage*" or "socio-economic* disadvantage*" or "socioeconomic inequit*" or "socio-economic inequit*" or "socioeconomic inequalit*" or "socio-economic inequalit*" or "disadvantaged group*" or "disadvantaged communit*" or "deprived area*" or "deprived communit*" or "deprived neighborhood*" or "deprived neighbourhood*" or immigra* or "hard-to-reach population*" or "health inequit*" or "low* education" or "pover*" or "vulnerable populations").mp. | "Low Socioeconomic Status"[Mesh]) OR "Healthcare Disparities"[Mesh] OR "Health Inequities"[Mesh] OR "Socioeconomic Factors"[Mesh] OR "Cultural Deprivation"[Mesh]OR "Emigrants and Immigrants"[Mesh] OR "Undocumented Immigrants"[Mesh] OR "Educational Status"[Mesh] OR "Poverty Areas"[Mesh] OR "Poverty"[Mesh] |
|  |  |  | 2: Screening | exp Screening/ or ("screening" or "screening programme").mp. | "Mass Screening"[Mesh] OR "Early Detection of Cancer"[Mesh] OR "diagnosis" [Subheading] OR "Diagnostic Screening Programs"[Mesh] OR "Direct-To-Consumer Screening and Testing"[Mesh] |
|  |  |  | 3: (Non)participation | ("participat*" or "nonparticip*" or "non-participat*" or "utiliz*" or "utilis*" or "uptake" or "up-take" or "take-up" or "takeup").mp. | "Patient Participation"[Mesh] OR "Community Participation"[Mesh] OR "Social Marginalization"[Mesh] OR "Refusal to Participate"[Mesh] OR "Delivery of Health Care"[Mesh] |
|  |  |  | 4: Barriers and facilitators | ("barrier*" or "obstacle*" or "facilitat*" or "enable*" or "motivat*" or "challeng*" or "resistan*").mp. | "Motivation" [Mesh] |
|  |  |  | 5: Chronic disease | exp Chronic Illness/ or chronic disease.mp. or exp Cardiovascular Disorders/ or exp Lung Disorders/ or exp Neoplasms/ or type 2 diabetes/ or exp Kidney Diseases/ | "Chronic Disease"[Mesh] OR "Chronic Disease Indicators"[Mesh] OR "Noncommunicable Diseases"[Mesh] OR "Neoplasms"[Mesh] OR "Early Detection of Cancer"[Mesh] OR "Cardiovascular Diseases"[Mesh] OR "Lung Diseases, Obstructive"[Mesh] OR "Pulmonary Disease, Chronic Obstructive"[Mesh] OR "Chronic Kidney Diseases of Uncertain Etiology"[Mesh] OR "Renal Insufficiency, Chronic"[Mesh] OR "Public Health"[Mesh] OR "Public Health Surveillance"[Mesh] |
| Web of Science | English language AND year=2013-search date (June 2023) | Topic | 1: People in vulnerable situations | ("low* socioeconomic status" or "low* socio-economic status" or "low* SES" or "low* income level*" or "low* health literacy" or "low* literacy" or "socioeconomic* disadvantage*" or "socio-economic* disadvantage*" or "socioeconomic inequit*" or "socio-economic inequit*" or "socioeconomic inequalit*" or "socio-economic inequalit*" or "disadvantaged group*" or "disadvantaged communit*" or "deprived area*" or "deprived communit*" or "deprived neighborhood*" or "deprived neighbourhood*" or immigra* or "hard-to-reach population*" or "health inequit*" or "low* education" or "pover*") or ("vulnerable populations") | Not applicable |
|  |  |  | 2: Screening | ("screen*") |  |
|  |  |  | 3: (Non)participation | ("participat*" or "nonparticip*" or "non-participat*" or "utiliz*" or "utilis*" or "uptake" or "up-take" or "take-up" or "takeup") |  |
|  |  |  | 4: Barriers and facilitators | ("barrier*" or "obstacle*" or "facilitat*" or "enable*" or "motivat*" or "challeng*" or "resistan*") |  |
|  |  |  | 5: Chronic disease | ("chronic disease" or "cancer") or (exp Chronic Disease/) or (exp Cardiovascular Diseases/) or (exp Lung Diseases/) or (Neoplasms/ ) or (exp Diabetes Mellitus, Type 2/) or (exp Renal Insufficiency, Chronic/) |  |
| CINHAL | English language AND year=2013-search date (June 2023) | Title OR Abstract OR Word in subject | 1: People in vulnerable situations | ("low* socioeconomic status" or "low* socio-economic status" or "low* SES" or "low* income level*" or "low* health literacy" or "low* literacy" or "socioeconomic* disadvantage*" or "socio-economic* disadvantage*" or "socioeconomic inequit*" or "socio-economic inequit*" or "socioeconomic inequalit*" or "socio-economic inequalit*" or "disadvantaged group*" or "disadvantaged communit*" or "deprived area*" or "deprived communit*" or "deprived neighborhood*" or "deprived neighbourhood*" or immigra* or "hard-to-reach population*" or "health inequit*" or "low* education" or "pover*") or ("vulnerable populations") | "Low Socioeconomic Status"[Mesh]) OR "Healthcare Disparities"[Mesh] OR "Health Inequities"[Mesh] OR "Socioeconomic Factors"[Mesh] OR "Cultural Deprivation"[Mesh]OR "Emigrants and Immigrants"[Mesh] OR "Undocumented Immigrants"[Mesh] OR "Educational Status"[Mesh] OR "Poverty Areas"[Mesh] OR "Poverty"[Mesh] |
|  |  |  | 2: Screening | (MH "Health Screening+") or ("screen*") | "Mass Screening"[Mesh] OR "Early Detection of Cancer"[Mesh] OR "diagnosis" [Subheading] OR "Diagnostic Screening Programs"[Mesh] OR "Direct-To-Consumer Screening and Testing"[Mesh] |
|  |  |  | 3: (Non)participation | ("participat*" or "nonparticip*" or "non-participat*" or "utiliz*" or "utilis*" or "uptake" or "up-take" or "take-up" or "takeup") | "Patient Participation"[Mesh] OR "Community Participation"[Mesh] OR "Social Marginalization"[Mesh] OR "Refusal to Participate"[Mesh] OR "Delivery of Health Care"[Mesh] |
|  |  |  | 4: Barriers and facilitators | ("barrier*" or "obstacle*" or "facilitat*" or "enable*" or "motivat*" or "challeng*" or "resistan*") | "Motivation" [Mesh] |
|  |  |  | 5: Chronic disease | (MH "Chronic Disease+") OR (MH "Pulmonary Disease, Chronic Obstructive+") OR (MM "Renal Osteodystrophy") OR (MH "Kidney Failure, Chronic+") | "Chronic Disease"[Mesh] OR "Chronic Disease Indicators"[Mesh] OR "Noncommunicable Diseases"[Mesh] OR "Neoplasms"[Mesh] OR "Early Detection of Cancer"[Mesh] OR "Cardiovascular Diseases"[Mesh] OR "Lung Diseases, Obstructive"[Mesh] OR "Pulmonary Disease, Chronic Obstructive"[Mesh] OR "Chronic Kidney Diseases of Uncertain Etiology"[Mesh] OR "Renal Insufficiency, Chronic"[Mesh] OR "Public Health"[Mesh] OR "Public Health Surveillance"[Mesh] |
| Embase | English language AND year=2013-search date (June 2023) | Topic | 1: People in vulnerable situations | ("low* socioeconomic status" or "low* socio-economic status" or "low* SES" or "low* income level*" or "low* health literacy" or "low* literacy" or "socioeconomic* disadvantage*" or "socio-economic* disadvantage*" or "socioeconomic inequit*" or "socio-economic inequit*" or "socioeconomic inequalit*" or "socio-economic inequalit*" or "disadvantaged group*" or "disadvantaged communit*" or "deprived area*" or "deprived communit*" or "deprived neighborhood*" or "deprived neighbourhood*" or immigra* or "hard-to-reach population*" or "health inequit*" or "low* education" or "pover*") or ("vulnerable populations") | Not applicable |
|  |  |  | 2: Screening | ("screen*") |  |
|  |  |  | 3: (Non)participation | ("participat*" or "nonparticip*" or "non-participat*" or "utiliz*" or "utilis*" or "uptake" or "up-take" or "take-up" or "takeup") |  |
|  |  |  | 4: Barriers and facilitators | ("barrier*" or "obstacle*" or "facilitat*" or "enable*" or "motivat*" or "challeng*" or "resistan*") |  |
|  |  |  | 5: Chronic disease | ("chronic disease" or "cancer") or (exp Chronic Disease/) or (exp Cardiovascular Diseases/) or (exp Lung Diseases/) or (Neoplasms/ ) or (exp Diabetes Mellitus, Type 2/) or (exp Renal Insufficiency, Chronic/) |  |
